# Microfluidic manipulation of magnetic flux domains in type-I superconductors: droplet formation, fusion and fission

**DOI:** 10.1038/s41598-017-11659-2

**Published:** 2017-09-21

**Authors:** G. R. Berdiyorov, M. V. Milošević, A. D. Hernández-Nieves, F. M. Peeters, D. Domínguez

**Affiliations:** 1 Qatar Environment and Energy Research Institute, Hamad Bin Khalifa University, Qatar Foundation, Doha, Qatar; 2 Departement Fysica, Universiteit Antwerpen, Groenenborgerlaan 171, B-2020 Antwerpen, Belgium; 3 Centro Atomico Bariloche and Instituto Balseiro, 8400 San Carlos de Bariloche, Rio Negro Argentina

## Abstract

The magnetic flux domains in the intermediate state of type-I superconductors are known to resemble fluid droplets, and their dynamics in applied electric current is often cartooned as a “dripping faucet”. Here we show, using the time-depended Ginzburg-Landau simulations, that microfluidic principles hold also for the determination of the size of the magnetic flux-droplet as a function of the applied current, as well as for the merger or splitting of those droplets in the presence of the nanoengineered obstacles for droplet motion. Differently from fluids, the flux-droplets in superconductors are quantized and dissipative objects, and their pinning/depinning, nucleation, and splitting occur in a discretized form, all traceable in the voltage measured across the sample. At larger applied currents, we demonstrate how obstacles can cause branching of laminar flux streams or their transformation into mobile droplets, as readily observed in experiments.

## Introduction

Moving superconducting vortices are known to be the main source for energy dissipation in current-carrying type-II superconductors, limiting their large scale energy-related applications. For that reason, much attention has been given in the past to hampering vortex motion by introducing arrays of artificial pinning centers in superconductors, nanoengineered in size and geometry for optimal vortex pinning and enhancement of maximal sustainable magnetic field and electric current in the superconducting state^1–17^. Pinning is also of importance in type-I superconductors, for example in defining the structure of the intermediate state (IS)^18,19^, which is a very rich study object and has received a revival of interest in recent years^20–32^. Contrary to type-II superconductors, the competition between the interface energy (that favors the formation of large normal domains) and the magnetic energy (that tends to form small normal domains) results in the formation of different spatially modulated IS structures in type-I superconductors^23,30^. There, flux tubes and lamellae are the most encountered shapes^18–20^, formation of which strongly depends on the size and shape of the samples^22–24^, as well as on the magnetic history of the system^18,19^.

Unlike Abrikosov vortices in type-II superconductors, each carrying a single flux quantum Φ_0_ = *hc*/2*e* ^33,34^, flux droplets in type-I superconductors may contain hundreds of flux quanta and are considered as building blocks for the IS flux patterns^35,36^. When driven by applied current, these flux structures can undergo different dynamic phases, where the motion of droplets can be periodic (with single or multiple periods) as well as chaotic^21^. Recent numerical simulations revealed that type-I flux droplets are always decomposed into singly-quantized fluxoids during dynamic interactions, reaffirming that one flux quantum is the smallest and fundamental building block of the IS in type-I superconductors^25^. Stationary flux droplets in type-I superconductors, with sizes down to singly quantized vortices, have been recently visualized in thick Pb films by scanning Hall probe microscopy^30^.

An important degree of complexity (and possibilities) is added to the problem when effect of pinning centers is considered, where, in addition to long-range repulsive magnetic interaction between the flux domains and the short-range attractive force due to interfacial tension, the interaction of the flux structures with the underlaying pinning landscape should be taken into account. To date, no theoretical study has addressed the response of type-I superconductors with artificial pinning centers to external electric and magnetic fields. In this context, of particular interest is the effect of such pinning centers on the topological transformations reported in recent experiments^18,19,36^. Furthermore, one wonders to which extent the flux droplets can be manipulated in the presence of pinning, possibly merged or split, using the nanoengineered pinning. In that respect, the experience of microfluidic community is very valuable, where similar manipulation of liquid droplets is of main interest^37^. In microfludic devices, the pressure gradient takes the role of the Lorentz force in superconductors. At the same time, the interaction of the droplets with obstacles is expected to be similar in the two (very remote) systems, due to the finite surface tension of the droplets, their ability to deform under hydrodynamic forcing, and pinch-off due to Rayleigh instability^38^ and pinning/depinning inertial deformations.

**Figure 1:**
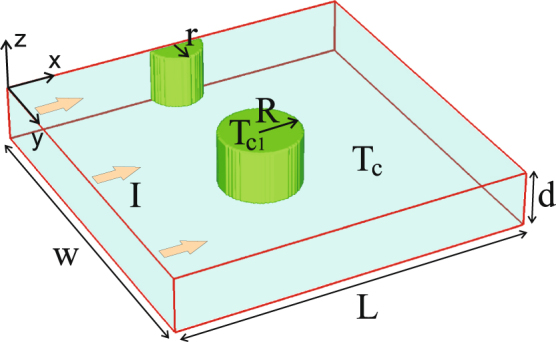
The considered system: a current-carrying superconducting slab (dimensions *w* × *L* × *d*) with an edge defect (radius *r*) and with a weakly (strongly) superconducting inclusion (depicted as circular with radius *R*) with critical temperature *T*_*c*1_ < *T*_*c*_ (*T*_*c*1_ > *T*_*c*_).

Therefore, in this work we explore the basic dynamics of IS flux structures in a current-carrying type-I superconducting slab, with one inclusion to serve as an obstacle for flux motion, as depicted in Fig. 1. For this purpose, we employ the time-dependent Ginzburg-Landau (TDGL) theory, which is the most suitable approach to describe the dynamics of the IS, taking into account the internal structure and elastic deformations of the flux domains^25,39,40^. Contrary to previous approaches available in the literature where domains were considered as singly-connected objects^41–44^, in our analysis the IS flux structures are allowed to dynamically decompose or merge due to encountered interactions in the sample^25^. We show that regardless of the size and the shape (tubular or laminar) of the domains, both their pinning and depinning processes at a trapping site occur in a discretized form with a single fluxoid at a time. Nevertheless, in terms of formation and manipulation of flux droplets, their merger and splitting, we show that the basic principles of microfludific passive fusion and fission can be successfully used. Just as size and manipulation of droplets in microfluidics is important for pharmaceutic applications, the one in type-I superconductors is applicable in hybrid devices where droplets are the localized sources of magnetic field that can e.g. trap and entangle spin and charge textures (see e.g. ref.^45^ and citing papers).

## Results

### Generation of flux droplets

In theory of superconductivity, the mechanism of flux entry is well studied^46–48^. In type-II superconductors, the conditions for vortex nucleation are reached at the sample edges when the velocity of the superconducting condensate there exceeds its critical value in increasing magnetic field. In type-I superconductors, the normal domains also nucleate at the sample edges, when the local magnetic field there exceeds the thermodynamic magnetic field *H*_*c*_. The minimal size of the created flux droplet in the sample is the one whose stray magnetic field reduces the field at the edge back below *H*_*c*_. In the presence of the applied electric current along the edges, as in our present case, the value of the current is directly related to the field at the sample edge, and hence dictates the size of the induced flux droplets. In addition, one can add a surface defect on the sample edge, where due to current crowding^49^ the local field conditions are changed^50,51^. In that case, the droplets nucleate exactly at the defect, and their size is controlled by the defect size. Therefore, in the present work, in order to facilitate the control of the nucleation and to further follow-up the flux droplets, we included an artificial defect of radius *r* at the edge for all simulations, where we consider that superconductivity is entirely suppressed (we take that the working temperature equals the critical temperature in the defect, see Fig. 1; this corresponds to setting the *T*_*c*_-nonuniformity coefficient *f*(*x*, *y*) = 0 inside the defect, see Methods). All results presented here are obtained without externally applied magnetic field.

**Figure 2:**
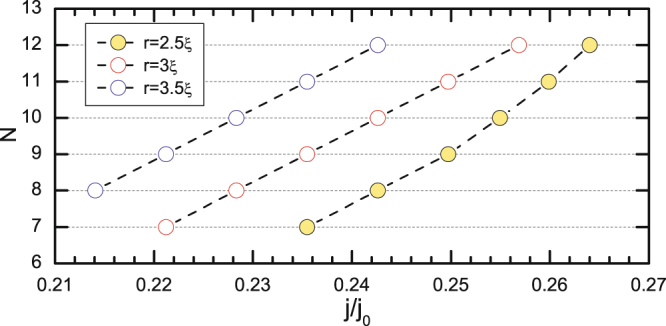
The size of the nucleated flux droplet (in units of flux quanta Φ_0_) as a function of the applied current density *j* and the radius of the edge defect *r*. Other parameters are *κ* = 0.4, *L* = *w* = 64*ξ*, and *d* = 12*ξ* (c.f. Fig. 1). Nonuniformity coefficient *f*(*x*, *y*) is set to zero inside the defect. Lower *κ* and larger *d* increase the size of the droplets, but reduce the range of current in which discrete droplets are formed.

As we showed in our previous work^25^, for sufficient applied current the flux penetrates the sample as singly-quantized vortices (as in the case of type-II superconductors) and the flux droplet is formed only at a distance ~10*ξ* inside the sample^44^. Figure 2 shows the dependence of the size of the flux droplet, upon pinch-off from the sample edge, as a function of the applied current and the size of the edge defect (for other parameters fixed as given in Sec. II). In other words, the defect acts exactly as a nozzle through which normal “fluid” is injected in the sample, whereas the applied current corresponds to the velocity of the superconducting “fluid” in the orthogonal direction. This cartoon is fully analogous to the pinching-off of a droplet at the end of the orifice of a capillary^52^, where applied current generates the magnetic field to create the droplet, and plays the role of gravity (i.e. determines the incoming stream), whereas the superconducting condensate provides the shearing force to ultimately cause droplet pinch-off. The size of the droplet is determined by the balance of the shearing and the interfacial forces, where latter depends also on the size of the orifice - in the present case, the size of the defect for the entry of flux droplets. As discussed above, flux droplets in superconductors are quantized objects, and for larger currents droplet formation becomes unfavorable - instead, a moving chain of singly-quantized vortices is formed, eventually turning into a continuous flow of the normal “fluid”.

### Droplet pinning

**Figure 3:**
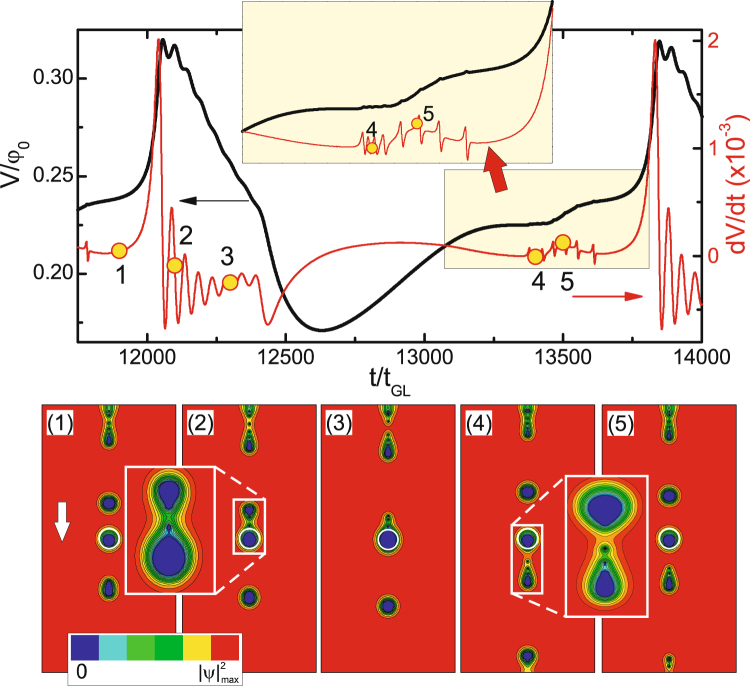
Voltage signal calculated near the pinning center (from *L*/4 to 3*L*/4) vs. time (*V*(*t*), thick black curve) and differential voltage-time (*dV*/*dt*, thin red curve, referred to right axis) characteristics of the sample (*κ* = 0.4, *L* = *w* = 64*ξ*, *d* = 12*ξ*, defects size *r* = 2.5*ξ*) for *R* = 3*ξ* and *f*(*x*, *y*) = 0. The applied current density is *j* = 0.246*j*_0_. Panels 1–5 show snapshots of the Cooper-pair density (in logarithmic scale) at time intervals indicated in the main panel, where the edge of the pinning center is shown by a white circle. The arrow in panel 1 indicates the direction of motion of flux droplets.

In what follows, we consider the interaction of the flux droplets with a nano-engineered pinning center or obstacle inside the sample. First we consider the case when the engineered center for droplet manipulation has a smaller critical temperature *T*_*c*1_ < *T*_*c*_ (i.e., *f*(*x*, *y*) < 1), acting as a pinning center for the incoming IS flux domains. Thick curve in the main panel of Fig. 3 shows the voltage vs. time characteristics of the sample with dimensions 64*ξ* × 64*ξ* × 12*ξ* and the GL parameter *κ* = 0.4 at *j* = 0.246*j*_0_ (please see Methods for definition of all units) for the size of the pinning center *R* = 3*ξ* and the nonuniformity coefficient *f*(*x*, *y*) = 0 there. At this value of the applied current the surface magnetic field exceeds the thermodynamic one near the surface defect, so that normal domains start nucleating, in this particular case, in the form of a “train” of flux droplets of same size (see panel 1)^53^. As we showed in our previous work^25^, flux penetrates the sample as singly-quantized vortices (as in the case of type-II superconductors) and the flux droplet is formed only at a distance ~10*ξ* inside the sample (panel 3)^44^. The same discretization process occurs during the expulsion of the flux droplet from the sample (see panels 4 and 5 of Fig. 3).

Both the penetration and the expulsion processes reflect in strong oscillations in the voltage signal across the sample, as discussed above^25^. To exclude such oscillations and focus only on the voltage response of the sample to the flux pinning and depinning in the central circular reservoir, we calculated the voltage signal in the area between *w*/4 and 3*w*/4 (shown in Fig. 3), thereby excluding the signal coming from the entrance and exit of vortices at the sample boundary. We find that the pinning of a flux droplet also occurs in a discretized form - one single flux quantum at a time (see panels 2 and 3 of Fig. 3) - leaving clear oscillatory traces in the local *V*(*t*) curve. Notice that the number of sequential voltage peaks exactly matches the size of the flux droplet (8 flux quanta in this particular case, c.f. with number of oscillatory peaks in the thin red curve in Fig. 3). After entire droplet is inside the pinning reservoir, a pronounced minimum develops in the voltage-time curve.

With time, the flux is depinned from the defect, again in a discretized form (see panel 4). Each individual vortex exit results in a visible feature in the differential voltage *dV*/*dt* (thin red curve in Fig. 3). Note that these features are observed on the background of increasing voltage, which is due to the motion of the new incoming flux droplet in the detection area (panel 5). The depinned single vortices merge into a droplet due to their positive interface energy (panel 5), and the final “break-off” of the domain takes place at the distance ~10*ξ* away from the defect, as in the case of flux penetration at the sample boundary. Resulting flux droplet is further driven by the Lorentz force and the voltage increases with time until the next droplet approaches the pinning center and the entire process repeats (see animated data of the time evolution of the Cooper-pair density in the Supplementary Video 1, showing the Φ_0_-discretized pinning and depinning of flux tubes by the defect). Notice that Fig. 3 shows just one period of the voltage oscillations.

**Figure 4:**
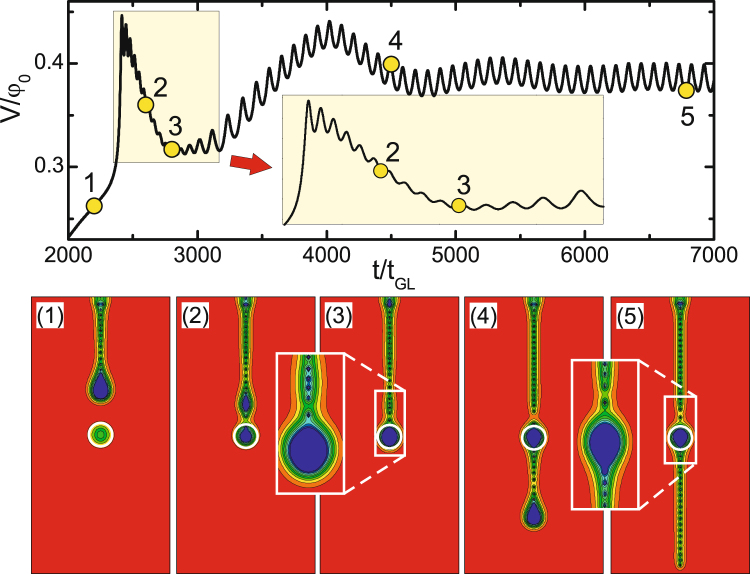
The same as in Fig. 3 but for a larger applied current density *j* = 0.275*j*_0_.

We here emphasize that the droplet contains the same number of flux quanta (in this particular case 8) before pinning and after depinning. This is driven by the long-range magnetic interaction between the droplets, requiring the uniformity of the “train” of moving droplets in the dynamic equilibrium. To illustrate this further, Fig. 4 shows the results for larger applied current, where tube formation is no longer energetically favorable and an elongated flux domain is formed. Due to its discrete dynamics, instead of the elongated IS stripe we observe a nearly continuous flow of single vortices towards the pinning reservoir (see panels 1 and 2 in Fig. 4). The flux fills the reservoir up to the “saturation” of the pinning center is reached (in this particular case 12Φ_0_), which also takes place in Φ_0_ discretized form (see associated voltage oscillations and the Supplementary Video 2 for animated data corresponding to Fig. 4). The voltage becomes entirely periodic at later time where each peak corresponds to consecutive single-flux quantum pinning and depinning. With further increasing current, the scenario remains unchanged, except for increasing frequency of the processes and the measured voltage.

### Droplet fusion

As a next step, we employ the accummulated knowledge about flux droplets to achieve flux accummulation and droplet fusion, referring again to the known principles of microfluidics. For that, we reverse engineer the pinning site, to make it act as a cummulative barrier for incoming flux droplets. For that, we employ defects which interact with the flux droplets repulsively, i.e., having *T*_*c*1_ > *T*_*c*_ (*f*(*x*, *y*) > 1). Such “anti-pinning” centers in superconductors can also be made geometrically, as pillars^54^ or using out-of-plane magnetized magnetic dots, when the applied field is antiparallel to the magnetic moment of the dots (see, e.g., refs^55–59^).

**Figure 5:**
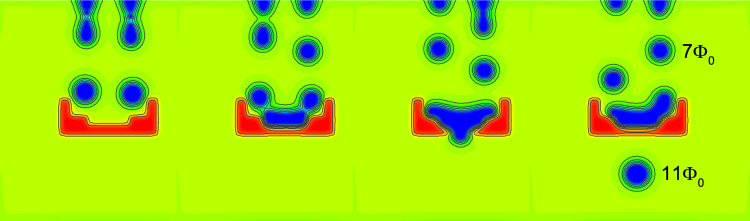
The sequence of snapshots of Cooper-pair density (taken at intervals of 10^3^ *t*_*GL*_), showing the fusion of droplets (of 7Φ_0_ each) generated at two faucets of radius 2.5*ξ* (with applied current density *j* = 0.24*j*_0_) and collected in a funnel of higher *T*_*c*_ [*f*(*x*, *y*) = 1.5]. The larger droplets (of 11Φ_0_ each) are periodically leaking out of the funnel. The full sequence of animated data is shown in Supplementary Video 3.

In particular, we use two “faucets” for incoming droplets, where size of the formed droplets for given applied current can be tuned by the size of the edge defect according to Fig. 2. In the path of those moving droplets, we make a repulsive wedge of a material with higher *T*_*c*_, that prevents further motion of droplets and guides them towards merger at the tip of the wedge. As shown in Fig. 5, this indeed works, and flux “liquid” accumulates in the wedge before the new droplets start to leak out at the tip of the wedge. In this particular example, the newly formed droplets contained 11Φ_0_ compared to initial 7Φ_0_. The size of the initial droplets can be tuned as already explained, and the size of the merged droplets will depend on the chosen *T*_*c*_ and precise shape of the barrier. This and similar geometries are readily used in microfluidics for passive mixing of droplets of different liquids. We again emphasize that in our system droplets are not entirely passive, due to long-range magnetic repulsion. Moreover, our droplets are quantized objects, and all interactions (however fast) are realized one flux quantum at a time.

### Droplet fission

In what follows, we find further use of repulsive barriers for flux droplets, but with an opposite intention compared to the previous example. Namely, contrary to droplet fusion, we will attempt to break the flux droplets by their interaction with a repulsive defect in their path of motion.

**Figure 6:**
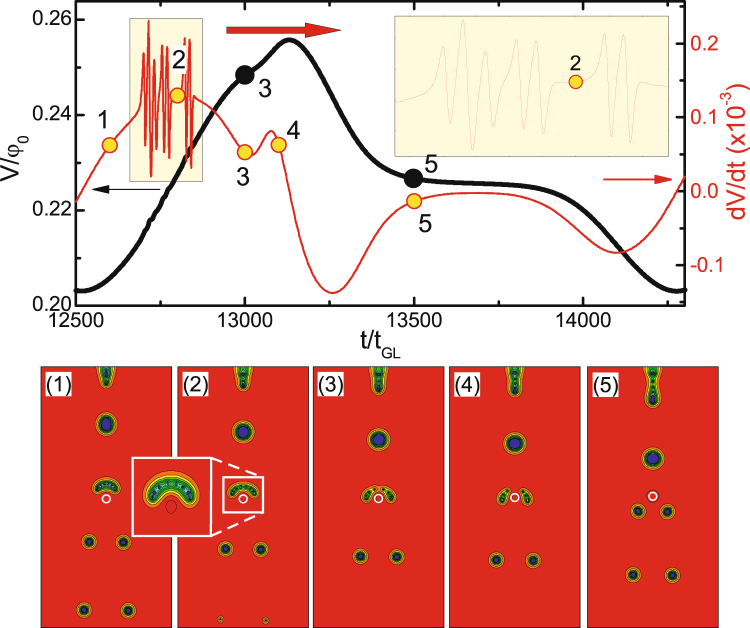
*V*(*t*) (thick black) and *dV*/*dt* (thin red) curves of the sample with dimensions 64*ξ* × 64*ξ* × 12*ξ* with a repulsive defect (*f*(*x*, *y*) = 2) of radius *R* = 1*ξ* for the applied current density *j* = 0.246*j*_0_. Panels 1–5 show the evolution of the intermediate state through the contourplots of |*ψ*|^2^ at time intervals indicated in the main panel.

As a representative example, we show in Fig. 6 the voltage vs. time characteristics of the sample (dimensions 64*ξ* × 64*ξ* × 12*ξ*) with a repulsive defect of radius *R* = 1*ξ* and nonuniformity coefficient *f*(*x*, *y*) = 2 in the middle of the sample. For the used applied current density *j* = 0.246*j*_0_, a train of flux droplets is formed at the surface defect (*r* = 2.5*ξ* and *f*(*x*, *y*) = 0) and the measured voltage shows periodic oscillations in time due to periodic entry of flux droplets into the detection area (panel 1). When the droplet reaches the defect, it stops and laterally splits in smaller (singly-quantized) units, in attempt to circumvent the defect. Eventually, in this particular case, two new droplets with equal number of flux quanta are formed, continuing their motion in two separate paths left and right of the defect (see panels 2–4 in Fig. 6). Supplementary Video 4 directly showplays the dynamics of this splitting process. The splitting leaves clear traces in oscillatory features of the *dV*/*dt* curve (see the inset in the main panel of Fig. 6), with sharp peaks corresponding to initial Φ_0_-splitting, a dip at the time of formation of two new droplets (points 3–4) and a noticeable plateau in the *V*(*t*) curve once those droplets continue their motion away from the defect (point 5). We found that for here used value of the *T*_*c*_-nonuniformity coefficient (*f*(*x*, *y*) = 2), the tubes can circumvent the defect without splitting only when the radius of the defect is very small, i.e. *R* < 1*ξ*.

**Figure 7:**
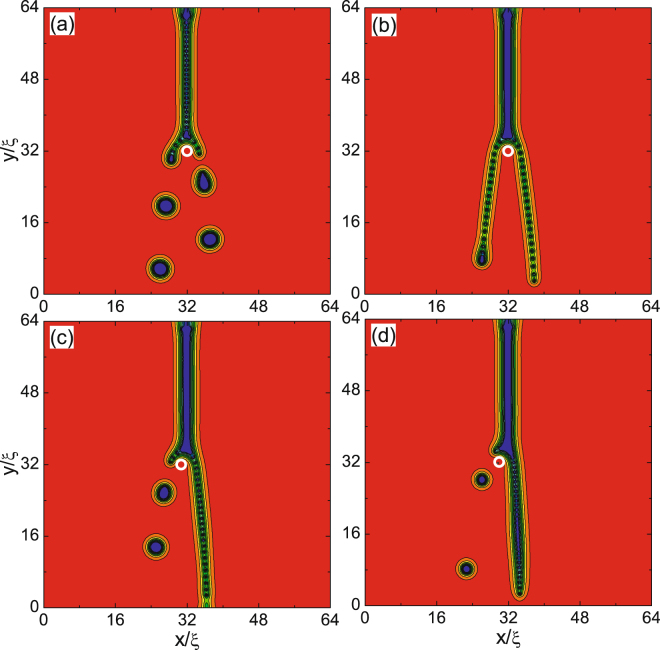
Snapshots of the Cooper-pair density in the sample of Fig. 6 at *j* = 0.29*j*_0_ (**a**) and *j* = 0.319*j*_0_ (**b**–**d**) at *t* = 30000*t*_*GL*_. The defect is located in the middle of the sample (**a**,**b**) and it is shifted by 1*ξ* (**c**) and 2*ξ* (**d**) in the -*x*-direction (i.e., in the direction of applied current).

With further increase of the applied current, the flux enters the sample at a larger pace and flows in the laminar formation, as shown in Fig. 7(a,b) [where we plot snapshots of the Cooper-pair density distribution in the same system, but for current values *j* = 0.29*j*_0_ (a) and *j* = 0.319*j*_0_ (b)]. When this normal strip reaches the defect it evolves into equal size and equally spaced droplets [see Fig. 7(a)]. Voltage response of the system in this regime is similar to the one shown in Fig. 4. Note that similar tubular phase is expected as the equilibrium pattern of the IS in the absence of driving current^18,19^. At higher driving forces, no such topological transformation takes place and only branching of laminar structures is observed, as shown in Fig. 7(b). The newly formed lamelae after the splitting contain less flux, hence their discrete nature may become more visible [as is the case in Fig. 7(b)].

Obviously, in the previous case we considered an ideallly symmetric situation, unattainable in any real sample. To address the effect of misalignment between the nucleation point of the flux droplet and the repulsive defect in their path of motion, we conducted simulations for the case when the defect is shifted by a small distance *dx* parallel to the applied current. Figure 7(c,d) show snapshots of the domain splitting for *dx* = 1*ξ* (c) and *dx* = 2*ξ* (d). It turns out that such a small displacement of the defect results in considerable changes in the evolution of the flux droplets: we observed a clearly asymmetric topological transformations of the laminar structures to combination of tubular and laminar phases [see Fig. 7(c,d)]. In practice, by varying *dx* between 0 and 2.8*ξ* at current *j* = 0.29*j*_0_, we could pinch off droplets of any size between 1 and 11Φ_0_ from the otherwise laminar shape of flux flow (see Supplementary Video 5 for direct visualization of pinching of flux droplets from the laminar flow, corresponding to Fig. 7).

Thus, repulsive defects in type-I superconductors can very effectively *split flux droplets into smaller ones*, *convert the laminar structures into mobile tubular droplets*, *or split off droplets of arbitrary size from the laminar flux flow*. These processes are analogous to passive manipulation of liquids in microfluidics, especially splitting of confined droplets at microfluidic junctions. Branching of the laminar normal domains in type-I superconductors is readily observed in the experiment (see, e.g., ref.^60^). We note once more that in superconductors all these processes are always *discretized* in individual vortices, and long-range magnetic interactions always play a role.

## Discussion

Droplet microfluidics is a well established field of manipulation of discrete fluid packets, essentially based on four major unit operations: droplet fusion, droplet fission, mixing in droplets, and droplet sorting. Each of these operations comes with challenges, thoroughly analyzed in related literature. In this paper, we point out the well-known fact that normal domains in the intermediate state of a type-I superconductor exhibit many analogies to fluid packets. We have principally focused on geometry-mediated passive interaction of droplets, to give only few examples of successful manipulation of magnetic flux domains in a type-I superconductor.

First we have discussed how the size of the edge defect in the presence of applied current plays the role of an orifice for injecting fluid droplets in the flow of another fluid. The span of realized droplet sizes as a function of the defect size and the applied current (shown in Fig. 2) can be further enlarged by e.g. applied magnetic field, or an adjusted shape of the edge (we considered a defect on a straight edge, whereas a concave shape of the sample would foster current crowding^49^, hence larger edge currents for the weaker applied one).

When it comes to droplet fusion in microfluidics, although it may seem straightforward, there is one key challenge involved in this process. Namely, in order for droplets to fuse, they must achieve temporal and spatial synchronization. In microfluidics, creative strategies have to be employed to synchronize droplets prior to fusion, both for passive and active droplet fusion systems. In superconductivity, synchronization of moving droplets of flux is not a challenge, it is a property directly achieved in the dynamic equilibrium. As a first step, we have shown that a pinning center of significant size (created by suppressed critical temperature, and/or blind hole, and/or magnetic dot on top) can act as a reservoir for accumulation of flux, in which droplets can be intermittently added and released. However, the synchronization of the droplets, enhanced by their long-range magnetic repulsion, leads to exactly same size of droplets going into and out of the reservoir. Still, this property is potentially useful for mixing (and possible entanglement) of the “information” carried by flux droplets, for example the confined spins in a DMS film underneath or on top of the superconductor^45^.

Further in the discussion of droplet fusion, we focused on the geometry-mediated passive microfluidic techniques. As a representative example, we chose to merge two channels of moving droplets by interaction with a nanoengineered “wall” in the shape of a funnel (e.g. region with higher *T*_*c*_, magnetic dot of an opposite polarity, geometrically higher barrier made of the same material, or similar). Here the size of both initial droplets is tunable (as explained above), and the resulting droplets depend on the properties and geometry of the funnel. This geometry-based manipulation is relatively easily scalable, and such carefully designed droplet operations allow for the multiplexing of a large number of droplets to enable complex assays fully analogous to ones used in biology and chemistry.

Droplet splitting is a reversed process, where we used an obstacle smaller than the droplet to split it in smaller ones. In e.g. ref.^61^, a microfluidic design for droplet splitting was proposed, where a large post near the middle of a microfluidic channel was used to induce droplet fission. By adjusting the position of this post in the microchannel, the ratio of sizes of daughter droplets can be changed. We have performed similar simulations, where the size of daughter droplets was tuned by shifting the obstacle with respect to the incoming flux flow. Here we went a step further, allowing for the nearly continuous flux flow in a shape of a lamella (basically a fully open “faucet”) from which droplets of arbitrary size can be detached by choosing the appropriate size and position of the obstacle.

As a major difference from microfluidic principles, flux droplets in type-I superconductors always interact in the form of *singly*-*quantized* vortices. All dynamical processes are Φ_0_-discretized, including entry (formation) of droplets at the sample edge, the subdivision of tubular configurations, conversion of lamellae into tubular phases, as well as branching of laminar patterns. Still, we hope that shown analogies will prove useful in understanding formation of flux patterns (in and out of equilibrium) often similar to those observed in various biological and physico-chemical systems^62^, but with underlying quantized nature bound to produce exciting differences.

## Methods

We perform numerical simulations on a current-carrying type-I superconducting slab with dimensions *L* × *w* × *d* (see Fig. 1), with nanoengineered defects of arbitrary shape - areas still superconducting but with critical temperature *T*_*c*1_ ≠ *T*_*c*_. For this system we solved the standard TDGL equations:

1 $$u\frac{\partial \psi }{\partial t}={(\nabla -i{\bf{A}})}^{2}\psi +(f(x,y)-{|\psi |}^{2})\psi ,$$

2 $${\kappa }^{2}{\rm{rot}}\,{\rm{rot}}\,{\bf{A}}={\rm{Re}}\,[{\psi }^{\ast }(-i\nabla -{\bf{A}})\,\psi ]-\frac{\partial {\bf{A}}}{\partial t}\equiv {\bf{J}},$$

where the spatially-dependent parameter *f*(*x*, *y*) = (1 − *T*/*T*_*c*1_(*x*, *y*))/(1 − *T*/*T*_*c*_) accounts for the *T*_*c*_-nonuniformity in the system: for *f*(*x*, *y*) < 1 (i.e., *T*_*c*1_ < *T*_*c*_) superconductivity is suppressed inside the defect (consequently it attracts the magnetic flux), whereas in the case of *f*(*x*, *y*) > 1 the defect is strongly superconducting (and interacts repulsively with the flux). This approach has been used previously to describe both static^63^ and dynamic^64^ properties of type-II superconductors with weakly superconducting inclusions. In Eqs (1 and 2), distance is scaled to the coherence length *ξ*(*T*), the vector potential **A** is in units of Φ_0_/2*πξ*(*T*), time is in units of the GL relaxation time *t*_*GL*_ = *πħ*/8*k*_*B*_(*T*_*c*_ − *T*)*u*, and voltage is scaled to *φ*_0_ = *ħ*/2*et*_*GL*_. Current density **J** is in units of *j*_0_ = *c*Φ_0_/(8*π* ^2^ *λ* ^2^ *ξ*). The material parameter *u*, which characterizes the ratio of the relaxation times of the phase and the amplitude of the order parameter *ψ*) is taken as *u* = 5.6 (Our conclusions are actually independent of the chosen value of *u*). Since our sample is thin enough to disallow vertical “branching” of domains (i.e., *d* $\ll$ 800(*ξ*-*λ*)^65^) both *ψ* and **J** may be averaged over the sample thickness^22^.

The magnetic screening is calculated via the inductance *B*_*z*_ = rot(**A**)|_*z*_ (see ref.^22^ and references therein):

3 $${B}_{z}({\bf{r}},z=\mathrm{0)}=\frac{1}{c}\,\int \,Q({\bf{r}},{\bf{r}}^{\prime} )\,g({\bf{r}}^{\prime} )\,{d}^{2}{\bf{r}}^{\prime} ,$$

where the scalar function *g*(**r**) is defined through ${\bf{J}}({\bf{r}})=\nabla \times \hat{{\bf{z}}}g({\bf{r}})$ and the kernel *Q* is chosen as $Q({\bf{r}})=4\pi \delta ({\bf{r}})-d/{[{|{\bf{R}}|}^{2}+{d}^{2}/4]}^{\mathrm{3/2}}$ ^22^. The boundary conditions (BC) are ${(\nabla -i{\bf{A}})\psi |}_{\perp }=0$ and *g* = 0. The transport current is introduced in the system through the BC setting the vector potential, rot **A**|_*z*_(*y* = 0, *L*) = ±*H*_*I*_, where *H*_*I*_ ≈ 2*πI*/*c* is the magnetic field induced by the injected current *I* ^66^. We solved Eqs (1–3) numerically, always starting from the fully superconducting state (i.e., |*ψ*| = 1). Subsequently we increased the current linearly over the time interval Δ*t* = 200*t*_*GL*_, from zero to its desired value, and then conducted simulations until the dynamically stable state was reached (typically up to total simulation time of 2 × 10^4^ *t*_*GL*_). The equations are solved self-consistently, in a centered time-difference scheme, with spatial grid discretization of 0.1*ξ* and a time step of 10^−4^ *t*_*GL*_. Such a small time step is necessary to ensure convergence of the system of equations in the presence of a strong self-induced field (as is the case in type-I superconductors) and fast moving flux domains (see e.g. ref.^67^).

## Electronic supplementary material

Supplementary information 1

Supplementary information 2

Supplementary information 3

Supplementary information 4

Supplementary information 5
